# A remarkable new species group of green seed beetles from genus *Amblycerus* Thunberg (Coleoptera, Chrysomelidae, Bruchinae), with description of a new Brazilian species

**DOI:** 10.3897/zookeys.401.6232

**Published:** 2014-04-14

**Authors:** Cibele Stramare Ribeiro-Costa, Marcelli Krul Vieira, Daiara Manfio, Gael J. Kergoat

**Affiliations:** 1 Laboratório de Sistemática e Bioecologia de Coleoptera, Departamento de Zoologia, Universidade Federal do Paraná, Caixa Postal 19020, 81531-980, Curitiba, Paraná, Brasil; 2 INRA-UMR CBGP (INRA/IRD/Cirad, Montpellier SupAgro), Campus International de Baillarguet, CS 30016, F-34988 Montferrier-sur-Lez, France

**Keywords:** Seed beetle, new species, taxonomy, key, Western hemisphere

## Abstract

Representatives of the subfamily Bruchinae (Coleoptera: Chrysomelidae) are usually small and inconspicuous, with only a few species drawing the attention. Here we deal with several unusually colored species of *Amblycerus* Thunberg, 1815, one of the two most diverse bruchine genera in the Western hemisphere. We define the *virens* group that consists of five species whose bodies are covered with a green vestiture, including one new for science, *Amblycerus medialis* Ribeiro-Costa, Vieira & Manfio, **sp. n.** (Type locality: Brazil: Pará, Rondônia). This study also provides redescriptions, diagnoses, comparative notes, illustrations, geographic distribution records and a key to the species in this group.

## Introduction

Bruchinae Latreille, commonly known as seed beetles, is one of the 13 subfamilies of Chrysomelidae (Bouchard et al. 2011). This subfamily encompasses more than 1700 species (Ribeiro-Costa and Almeida 2012) that are distributed worldwide. In the Western hemisphere, two genera, *Amblycerus* Thunberg, 1815 (Amblycerini: Amblycerina) and *Acanthoscelides* Schilsky, 1905 (Bruchini: Acanthoscelidina), stand out as the most hyperdiverse genera (Kingsolver 1990, Ribeiro-Costa 1999), the first with 340 species (Kingsolver 1990) and the second with more than 100 species (Romero et al. 1996, Ribeiro-Costa 2000). For *Acanthoscelides*, several molecular analyses indicate that the genus is likely paraphyletic (Kergoat and Silvain 2004, Kergoat et al. 2005, 2008), which is not surprising considering the lack of clear diagnostic characters for the genus (Borowiec 1987, Kergoat and Silvain 2004). On the contrary, a morphological cladistic analysis of *Amblycerus* sampled in the United States and Mexico (Romero et al. 2002) suggests that the genus *Amblycerus* is possibly monophyletic.

Members of *Amblycerus* are well defined and easily recognized by their subovate body, shallowly emarginate eyes, hind tibia without prominent lateral carinae, and the presence of two apical spurs on hind tibia (Romero et al. 1996, Kingsolver 2004). Though most species for which host plants are known usually develop on Fabaceae (Romero et al. 1996, 2002), several *Amblycerus* species are quite remarkable because they are associated with other plant families (Romero et al. 1996, 2002). In total, at least 13 distinct plant families have been thus recorded for the genus *Amblycerus*, a pattern that contrasts with most bruchine genera that are only associated with one or a few host plant families (Borowiec 1987). Another interesting feature of *Amblycerus* is the unusual coloration pattern of a few species. In general seed-beetles have a black, yellow or reddish non-metallic body (Borowiec 1987). Their vestiture is more or less dense, and is usually not made of conspicuous colors. Among *Amblycerus*, several species clearly depart from this pattern as they exhibit a conspicuous green vestiture. The first species with such an unusual coloration pattern, *Amblycerus virens* (Jekel, 1855), was described in 1855 from French Guiana. Following a revisional work initiated almost 20 years ago as a part of a thesis on Brazilian *Amblycerus* species (Ribeiro-Costa 1995), three other species were later described in 1998: *Amblycerus virescens* Ribeiro-Costa, 1998 *Amblycerus viridans* Ribeiro-Costa, 1998 and *Amblycerus viridis* Ribeiro-Costa, 1998. At that time, phenetic analyses were used to place these four species in a distinct species group (Ribeiro-Costa 1995), but a clear formalization of the corresponding species group (*virens* group) is lacking to date. As these species with a green vestiture do not occur in the United States and Mexico they were also not included in the morphological cladistic analysis of Romero et al. (2002).

To advance in the taxonomy and systematics of the *virens* group we propose a revision of the entire species group, including a redescription of *Amblycerus virens* and a description of a new *Amblycerus* species that also harbors this unusual green vestiture. We also provide an identification key, geographic distribution data and a diagnosis for the group based on comparisons of morphological characters used at group level in previous taxonomic and cladistic studies (Romero et al. 1996, 2002). All these species possibly form a natural group, but comprehensive phylogenetic studies (with a dense sampling of *Amblycerus* species) are definitely required to precise this hypothesis.

## Material and methods

The material examined was loaned from museums/collections listed below (acronyms of museums/collections and curators’ names are also provided).

CNCI Canadian National Collection of Insects, Ottawa, Canada, (A. E. Davies);

CMNH Carnegie Museum of Natural History, Pittsburgh, United States, (R. Davidson);

DZUP Coleção de Entomologia Pe. J.S. Moure, Departamento de Zoologia, Universidade Federal do Paraná, Curitiba, Paraná, Brazil, (C. S. Ribeiro-Costa);

FSCA Florida State Collection of Arthropods, Gainesville, Florida, United States, (M. C. Thomas);

FIOC Fundação Instituto Oswaldo Cruz, Rio de Janeiro, Rio de Janeiro, Brazil, (J. Costa);

MZSP Museu de Zoologia da Universidade de São Paulo, São Paulo, Brazil, (S. Casari);

MPEG Museu Paraense Emílio Goeldi, Belém, Pará, Brazil, (O. T. Silveira);

BMNH The Natural History Museum, London, United Kingdom, (S. Shute);

USNM United States National Museum of Natural History, Washington D.C., United States, (A. Konstantinov and E. Roberts).

Most characters were observed from dry pinned insects. Male genitalia were studied following Manfio et al. (2013), except for *Amblycerus virens* for which we followed Ribeiro-Costa (1998). Colored images of the external morphology were captured with a LEICA DFC 500 digital camera attached to a LEICA MZ16 stereomicroscope, and subsequently processed using Auto-Montage Pro (Syncroscopy) image processing software of the “TAXon line- Rede Paranaense de Coleções Biológicas” at the Departamento de Zoologia, Universidade Federal do Paraná (UFPR). The terminology adopted was that of Ribeiro-Costa and Silva (2003) except for some thoracic and abdominal sclerites for which we followed Lawrence et al. (2010). Measurements were obtained using AXIOVISION version 4.8.2.0 over images captured with a SONY CYBER-SHOT DSC W350 digital camera coupled in the stereoscopic microscope CARL ZEISS DISCOVERY version 8. Measurements of body parts (body length and width, ocular index and *sinus*, postocular lobe, antennomeres length and width) were carried out on one randomly chosen specimen of each species following traits highlighted in Ribeiro-Costa (1998: 631, Figs 1, 2, 3, 5 and 8). The following abbreviations were used: BL, body length (from anterior margin of pronotum to elytra apex) and BW, body width (the largest width on the subapical region of the elytra).

## Results and discussion

The *virens* species group consists of five species: *Amblycerus virens* (Jekel, 1855), *Amblycerus virescens* Ribeiro-Costa, 1998, *Amblycerus viridans* Ribeiro-Costa, 1998, *Amblycerus viridis* Ribeiro-Costa, 1998 and *Amblycerus medialis* Ribeiro-Costa, Vieira & Manfio, sp. n. It can be distinguished from other *Amblycerus* species groups by combinations of characters that are listed below in the diagnosis.

### Group *virens*

Figs 1–12

**Figure 1–6.**
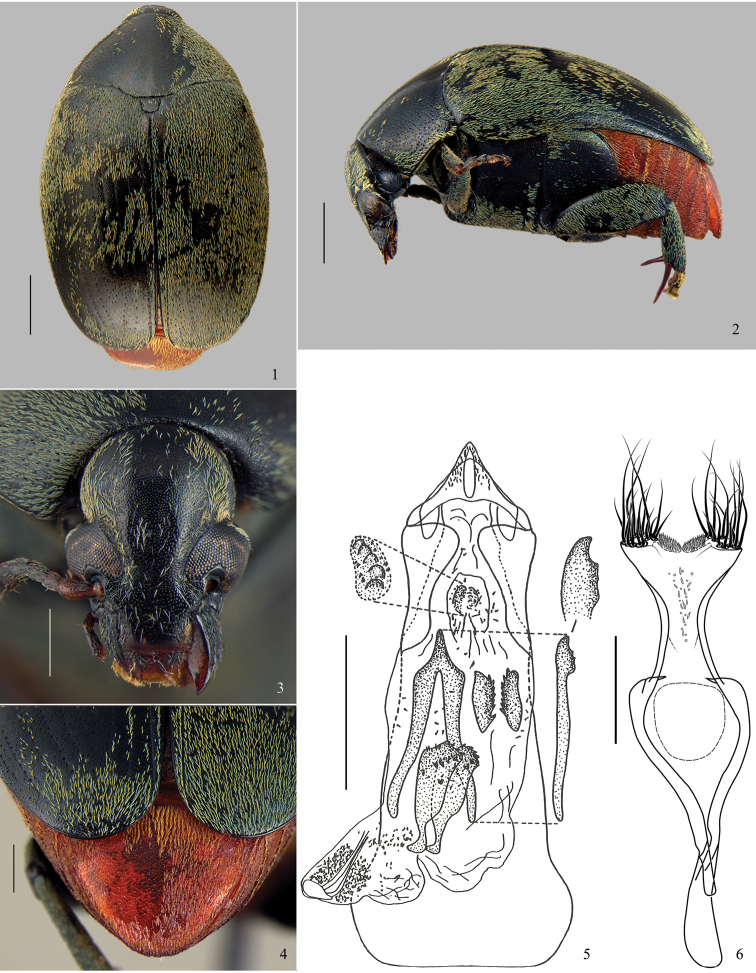
*Amblycerus virens* (Jekel, 1855), specimen male: **1** dorsal **2** lateral **3** head **4** pygidium **5** median lobe of male genitalia **6** tegmen of male genitalia. Scale bars = 1.0 mm (Figs **1–2**); scale bars = 0.5 mm (Figs **3–4, 6**); scale bar = 0.2 mm (Fig. **5**).

**Figure 7–12.**
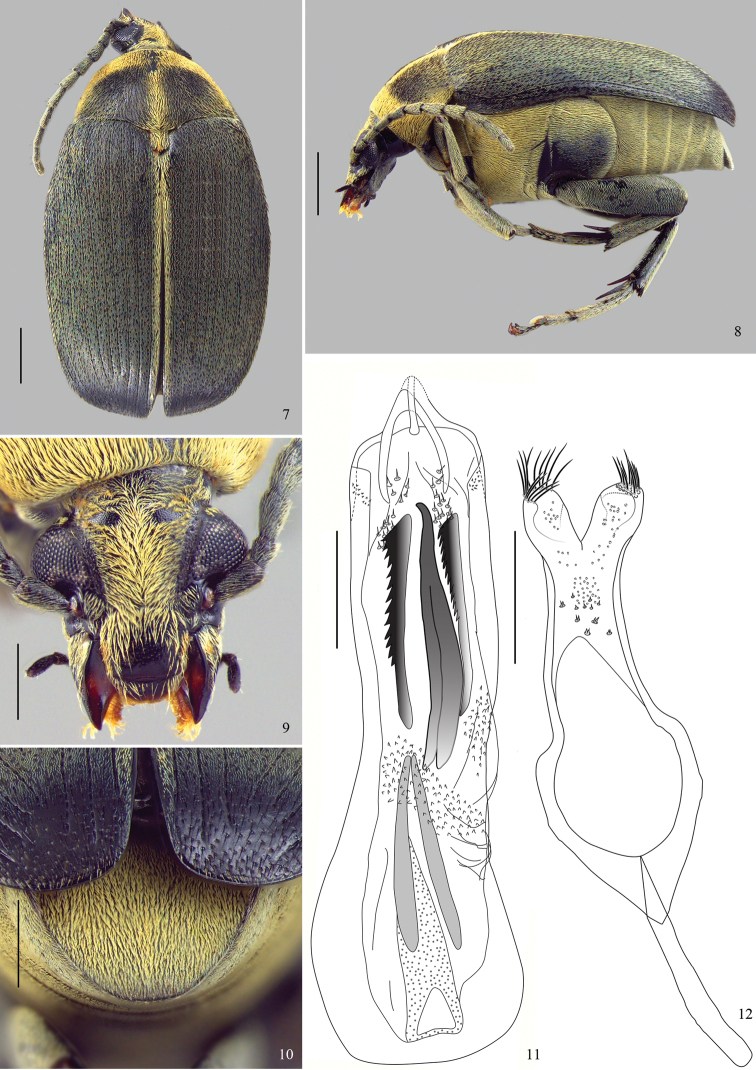
*Amblycerus medialis* Ribeiro-Costa, Vieira & Manfio, sp. n., holotype male: **7** dorsal **8** lateral **9** head **10** pygidium **11** median lobe of male genitalia **12** tegmen of male genitalia. Scale bars = 1.0 mm (Figs **7–8**); scale bars = 0.5 mm (Figs **9–12**).

**Diagnosis.** Most of dorsum covered with a green vestiture (Figs 1, 7); pygidium with yellowish setae homogeneously distributed (Figs 4, 10). Head covered with fine and dense punctures, without frontal carina (Figs 3, 9); frontoclypeal suture indistinct (Fig. 3). Disc of pronotum semicircular (Figs 1, 7), with background covered with fine and dense punctures; lateral carina almost reaching the anterior margin of pronotum (Fig. 2); cervical sulcus absent; prosternal process not expanded beyond anterior coxae, slightly constricted between coxae. Metepisternum without transverse, fusiform, curved and striate file; metepisternal sulcus forming right angle. Metaventrite with moderately coarse and sparse punctures; median sulcus one-half as long as metaventrite. Pygidium with apical margin rounded (Figs 4, 10). Internal sac of male genitalia at the median region with a pair of blade sclerites with serrate margin and a wishbone-shaped unpaired sclerite (Figs 5, 11).

**Comparative notes.** Within the *virens* group, *Amblycerus virens*, *Amblycerus virescens*, *Amblycerus viridans* and *Amblycerus viridis*, share more morphological similarities to each other than with *Amblycerus medialis* Ribeiro-Costa, Vieira & Manfio, sp. n. The most obvious difference between them is the fact that for *Amblycerus virens*, *Amblycerus virescens*, *Amblycerus viridans* and *Amblycerus viridis*, the pubescence on pronotum and elytra is not variegated and does not present stripes.

In comparison with other *Amblycerus* species, it is worthy to note that *Amblycerus medialis* presents two long, serrate blades in the internal sac of male genitalia. Interestingly, these serrate blades (character 24(1), pg. 7, Romero et al. (2002)) are also found in two species (*Amblycerus barcenae* and *Amblycerus pictus*) of the *marmoratus* clade (Romero et al. 2002). The broader blades of *Amblycerus viridis*, *Amblycerus virescens* and *Amblycerus viridans* are more similar with those of many other Brazilian species studied by Ribeiro-Costa (1995). *Amblycerus viridis*, *Amblycerus viridans* and *Amblycerus virens* also share the presence of two plates with small tubercles on the dorsal surface of the internal sac of male genitalia (character 25(1), pg. 7, Romero et al. (2002)) with the clade *anosignatus* (composed of *Amblycerus anosignatus*, *Amblycerus chiapas* and *Amblycerus guerrerensis*). The issue of determining whether these morphological similarities are homoplastic or not is complex, and will clearly benefit from results of future phylogenetic analyses of molecular datasets.

**Sexual dimorphism.** Sexual dimorphism was not observed even in the shape of the apex of the last abdominal ventrite.

**Geographical distribution.** Neotropical region, although the species from this group are more commonly distributed between the North of French Guiana to Midwest Brazil.

**Host plants.** This species group does not have known host plants records.

### Key to males of *virens* group

**Table:** 

1	Eyes prominent laterally (Ribeiro-Costa 1998: 636, Figs 3, 9, 16), metaventrite protuberant between mid coxae in lateral view (Ribeiro-Costa 1998: 636, Figs 2, 17)	2
1’	Eyes flat laterally (Ribeiro-Costa 1998: 631, Fig. 1) metaventrite flat between mid coxae in lateral view (Ribeiro-Costa 1998: 631, Fig. 4)	3
2 (1)	Pronotum and elytra with mid strip of vestiture (Fig. 7), antennae serrate from 4 to 10 antennomere (Fig. 8), ocular index: 2,04–2,22	*Amblycerus medialis* Ribeiro-Costa, Vieira & Manfio, sp. n.
2’	Pronotum with uniform pattern of vestiture, lacking stripes (Fig. 1), antennae moderately serrate from 5 to 10 antennomere (Ribeiro-Costa 1998: 631, Fig. 3), ocular index: 2.41–3.43	4
3 (1’)	Median lobe of male genitalia with wishbone sclerite as long as the blade sclerites (Ribeiro-Costa 1998: 632, Fig. 9)	*Amblycerus virescens* Ribeiro-Costa, 1998
3’	Median lobe of male genitalia with wishbone sclerite more than half of the length of the blade sclerites (Ribeiro-Costa 1998: 634)	*Amblycerus viridans* Ribeiro-Costa, 1998
4 (2’)	Median lobe of male genitalia with blade sclerites longer than wishbone sclerite, denticulate from the apex to half its length (Ribeiro-Costa 1998: 636, Fig. 19)	*Amblycerus viridis* Ribeiro-Costa, 1998
4’	Median lobe of male genitalia with blade sclerites about one half of wishbone sclerite length, with denticles restricted to subapical region (Fig. 5)	*Amblycerus virens* (Jekel, 1855)

### Amblycerus virens

(Jekel, 1855)

http://species-id.net/wiki/Amblycerus_virens

Figs 1–6

Spermophagus virens Jekel, 1855: 33 (holotype, type locality: French Guiana; description, distribution); Gemminger and Harold 1873: 3219 (catalog, distribution); Pic 1913: 63. (catalog, distribution).
Amblycerus virens : Blackwelder 1946: 763 (catalog, new combination); Udayagiri and Wadhi 1989: 16 (catalog); Ribeiro-Costa 1998: 633 (citation).

#### Redescription.

BL: 5.6 mm; BW: 3.84 mm

Integument (Figs 1–4). Body mostly black, mouth parts and antennomeres 1 and 2 brown to yellowish, apical spurs of hind tibiae reddish brown, pygidium and abdomen rufous with golden shine.

Vestiture (Figs 1–4). Pronotum, elytra and thorax covered with greenish setae, abdomen and pygidium with yellowish setae, both not variegated.

Head (Fig. 3). Covered with fine and dense punctures. Frons without frontal carina. Eye finely faceted, moderately prominent laterally. Ocular index: 3.11; ocular *sinus*: 0.63; postocular lobe 0.33 the eye length. Antenna not reaching anterior margin of hind coxa; moderately serrate from antennomeres 5-10 (Ribeiro-Costa 1998: 631, Fig. 3); from 5 to11 antennomeres 1.33 wider than long; last antennomere with truncate apex. Frontoclypeal suture indistinct. Clypeus covered with fine and dense punctures except in narrow band on apical portion. Labrum with few fine punctures on basal margin.

Prothorax. Pronotum semicircular; covered with fine and dense punctures, moderately coarse punctures intermixed on lateral areas (Fig. 1). Lateral carina almost reaching the anterior margin of pronotum (Fig. 2). Cervical sulcus absent. Prosternal process not expanded beyond anterior coxae; flat and slightly constricted between coxae.

Mesothorax and metathorax. Scutellum as long as wide; round or unidentate at apex (Fig. 1). Elytron with striae 1 and 10 moderately impressed; 2, 3, 8 and 9 weakly impressed until the third apical region of elytron then only isolated punctures representing striae; 4–7 striae formed only by isolated punctures; 4 and 5 anastomosed before the fusion of 6+7. Interstriae with moderately coarse and sparse punctures. Metaventrite moderately protuberant (Fig. 2) with moderately coarse and sparse punctures; median sulcus one-half as long as metaventrite. Metepisternum with moderately coarse and sparse punctures; metepisternal sulcus forming straight angle, with transverse axis straight and not reaching lateral margin of metepisternum. Mid coxae lower than anterior coxae, in lateral view (Fig. 2). Hind femur about 2.5 times longer than wide. Hind coxae with moderately coarse and sparse punctures. Hind tibia lateral spur about 1.5 times the length of median spur; 1-tarsomere about 1.5 the length of the lateral spur and 2.5 times the median spur.

Abdomen (Fig. 4). Ventrites with moderately coarse and dense punctures; last ventrite as long as 4-ventrite. Pygidium one-third covered by the elytra; apical margin rounded, with moderately coarse and dense punctures.

Male genitalia. Median lobe (Fig. 5) about 4.15 times its widest at apical region; ventral valve with lateral margins straight; dorsal valve with lateral margins concave and acuminate apex. Internal sac (Fig. 5) in the apical region without anterior sclerites; a pair of tuberculate median sclerites; a pair of ovoid and denticulate posterior sclerites. Median region with a pair of sinuous blade sclerites, sinuous at base and denticulate at apex; a long wishbone sclerite, about two times longer than the blade sclerites, curved and denticulate at apex and with stems moderately separated. Basal region of sac without sclerites; apical and median regions with several spines. Tegmen (Fig. 6) slightly emarginated between lateral lobes expanded.

#### Type material.

Syntype studied by the first author and deposited in BMNH, sex undetermined, labels: ‘Type H. T.’ [round, white with red margin]; ‘Cayenna’ [white]; ‘ex. Mus. W. W. S.’ [white]; ‘Type’ [white]; ‘53272’ [white]; ‘Fry Coll. 1905. 100’ [white]; ‘*Spermophagus virens* Dj. n. sp. Cayen’; ‘SYNTYPE/Spermophagus/virens Jekel, 1855/Ribeiro-Costa, C. S.’

#### Notes.

Jekel (1855) described the material quoted by Dejean (1837) but in his description, he did not specify how many specimens were studied. Therefore the exemplar received from BMNH, from the locality quoted in the original description, is a type specimen and regarded as a syntype.

#### Additional material.

**BRAZIL:** Amazonas: 1 male specimen, São Gabriel, Rio Negro, 9.X.1927, J.F. Zikán, (FIOC). Pará: 1 male specimen, Santarém, VII.1919, S. M. Klages(CARN). **FRENCH GUIANA:** Bélvédère de Saül: 1 male specimen, Mont Itoupé, 30.III.2010, P. H. D. leg. (DZUP).

#### Distribution.

Brazil (Amazonas and Pará), Fench Guiana.

#### Comparative notes.

*Amblycerus virens* differs from the other species of the group by the length of the lateral spur of hind tibia (2.4 times the length of median; Fig. 2) (for other species the length of the lateral spur of hind tibia is less than 1.85 times the length of median); the internal sac of male genitalia has small blade sclerites (Fig. 5) (other species in the group have long blade sclerites).

This species shares with *Amblycerus viridis* and *Amblycerus medialis* the prominent eyes (Figs 3, 9), postocular lobe narrower and metaventrite protuberant in lateral view (Figs 2) but the male genitalia do not show closer similarities among these species. *Amblycerus virens* and *Amblycerus virescens* share a long wishbone sclerite comparing to the blades in the internal sac of male genitalia (Fig. 5; Ribeiro-Costa 1998: 632. Fig. 9).

### Amblycerus virescens

Ribeiro-Costa, 1998

http://species-id.net/wiki/Amblycerus_virescens

Amblycerus virescens Ribeiro-Costa, 1998: 630 (original description, holotype, type locality: Brazil, Amazonas). Ribeiro-Costa 1998: 631–632, Figs 1–10. Detailed description and information of type material are in Ribeiro-Costa (1998).

#### Distribution.

Brazil (Amazonas, Amapá and Goiás).

#### Comparative notes.

This species can be distinguished from all other of *virens* group by the internal sac of male genitalia that has a pair of subtriangular sclerites with denticulate margins (Ribeiro-Costa 1998: 632. Fig. 9) (*Amblycerus virens*, *Amblycerus viridis* and *Amblycerus viridans*, have a pair of sclerites with small rounded protuberances) (Fig. 5; Ribeiro-Costa 1998: 634, 636. Figs 14, 19); *Amblycerus medialis* Ribeiro-Costa, Vieira & Manfio, sp. n. absent (Fig. 11)).

*Amblycerus virescens* and *Amblycerus viridans* have no salient eyes (Ribeiro-Costa 1998: 631. Fig. 1), postocular lobe long, and metaventrite not protuberant (Ribeiro-Costa 1998: 631. Fig. 4). However, comparisons of male genitalia indicate that *Amblycerus virescens* is similar to *Amblycerus virens* because they both have a wishbone sclerite that is longer than the blades (Fig. 5; Ribeiro-Costa 1998: 632. Fig. 9).

### Amblycerus viridans

Ribeiro-Costa, 1998

http://species-id.net/wiki/Amblycerus_viridans

Amblycerus viridans Ribeiro-Costa, 1998: 633 (original description, holotype, type locality: Brazil, Mato Grosso). Ribeiro-Costa 1998: 634, Figs 11–15. Detailed description and information of type material are in Ribeiro-Costa (1998).

#### Distribution.

Brazil (Mato Grosso).

#### Comparative notes.

*Amblycerus viridans* differs from all other species in the group by the structure of the internal sac of male genitalia, which includes a pair of sclerites formed by dense denticles (Ribeiro-Costa 1998: 634, Fig. 14: EPL) (character absent in *Amblycerus virescens*, *Amblycerus viridis*, *Amblycerus virens* and*Amblycerus medialis*). This species is similar to *Amblycerus viridis* because they both have a wishbone sclerite that is shorter than the blades; in addition both species also share the presence of a pair of slender denticulate sclerites on median region (Ribeiro-Costa 1998: 634, Fig. 14: EPC). Additional information on external morphological similarities is also presented in the section dedicated to *Amblycerus virescens*.

### Amblycerus viridis

Ribeiro-Costa, 1998

http://species-id.net/wiki/Amblycerus_viridis

Amblycerus viridis Ribeiro-Costa, 1998: 635 (original description, holotype, type locality: Brazil, Mato Grosso). Ribeiro-Costa 1998: 636, Figs 16–19. Detailed description and information of type material are in Ribeiro-Costa (1998).

#### Distribution.

Brazil (Mato Grosso).

#### Comparative notes.

*Amblycerus viridis* differs from the other species in the group by its shorter hind femur, which is 2.32 times longer than its width (in others species the ratio is superior to 2.5 times). Additional information on external and internal similarities is also presented in the sections dedicated to *Amblycerus virens* and *Amblycerus viridans*.

### Amblycerus medialis

Ribeiro-Costa, Vieira & Manfio sp. n.

http://zoobank.org/D819155A-D242-4BE0-813B-B5B258DD4554

http://species-id.net/wiki/Amblycerus_medialis

Figs 7–12

#### Description.

BL: 6.3 mm; BW: 3.78 mm

Integument color (Figs 7–10). Body black except mouth parts brownish; apical spurs of hind tibiae brownish to black.

Vestiture (Figs 7–10). Pronotum with a predominantly green vestiture but also with yellowish setae on the anterior margin, lateral areas and median line; elytra with a predominantly green vestiture but also with yellowish setae on 1 interstria; thorax and abdomen covered with pale yellowish setae.

Head (Fig. 9) covered with fine and dense punctures. Frons without frontal carina. Eyes moderately faceted, strongly prominent laterally. Ocular index: 2.23; ocular *sinus*: 0.78; postocular lobe 0.34 the eye length. Antennae not reaching anterior margin of hind coxa (Fig. 8); serrated from 4 to 10 antennomeres; from 3 to 11 antennomeres 1.94 longer than wider; 11 antennomere with truncate apex (Fig. 8). Frontoclypeal suture indistinct. Clypeus covered with fine and dense punctures except on a narrow band on apical portion. Labrum with few fine punctures on basal margin.

Prothorax (Fig. 7). Pronotum semicircular; covered with fine and dense punctures, moderately coarse punctures intermixed all over pronotum. Lateral carina complete, almost reaching the anterior margin of pronotum. Cervical sulcus absent. Prosternal process longer than anterior coxae, gently arched between coxae and slightly constricted between coxae.

Mesothorax and Metathorax. Scutellum longer than wide with tridentate apex. Elytron with striae moderately impressed, not fused apically. Interstriae with moderately coarse and dense punctures (Fig. 7). Metaventrite slightly protuberant with moderately coarse and sparse punctures; median sulcus one-half as long as metaventrite. Metepisternum without punctures; metepisternal sulcus forming straight angle, with transverse axis straight and reaching lateral margin of metepisternum. Mid coxae lower than anterior coxae, in lateral view (Fig. 8). Surface of hind coxae without punctures. Hind femur 3 times longer than wide. Hind tibia lateral spur about 1.5 times the length of median spur, and 1-tarsomere about 1.5 the length of lateral spur and 2.5 times the median spur.

Abdomen (Fig. 10). Ventrites finely punctulate, the last about 2 times wider than the 4-ventrite; pygidium one-half covered by the elytra, with apical margin rounded, with fine punctures.

Male genitalia (Figs 11–12). Median lobe (Fig. 11) about 5.43 times its widest at apical region; ventral valve with lateral margins concave, dorsal valve with lateral margins straight. Internal sac (Fig. 11) in the apical region without sclerites. Median region with a pair of straight blade sclerites, one side denticulate; wishbone sclerite as long as blade sclerites, curved at apex and stems moderately separated. Basal region with a sclerite with long stems gradually approximated. Apical and median regions with several spines and denticles. Tegmen (Fig. 12) deeply emarginated, about 0.35 times the length of the expanded lateral lobes.

#### Type material.

Holotype deposited in FSCA, male, with labels: ‘BRAZIL: Rondônia 62/ km SW Ariquemes, nr/ Fzda. Rancho Grande/ 8-20-XI-1994 JE Eger/ MV & Black Lights’[white, printed in black]; ‘FSCA’ [green, printed in black]; ‘HOLOTYPE/ Amblycerus medialis/ Ribeiro-Costa, Vieira & Manfio, / [white with red margin, printed in black] (FSCA). 1 paratype deposited in CNCI, female, with labels: ‘BRAZIL, Pará ♀/ Faz. Taperinha/ XI-19-22-1969/ JM & BA Campbell’ [white, printed in black]; ‘CNC’ [white with green line in the middle, printed in black]; ‘PARATYPE/ Amblycerus medialis/ Ribeiro-Costa, Vieira & Manfio/ [white with yellow margin, printed in black] (CNCI).

#### Distribution.

Brazil (Pará and Rondônia).

#### Comparative notes.

*Amblycerus medialis* can be easily separated from others species in the group by the presence of yellow pubescent stripes on the pronotum and elytra (Fig. 7) (others species are exclusively with a green vestiture); antennomeres about 2 times as long as wide (Figs 7–8) (others wider than long).

Additional information on external and internal similarities is also presented in the sections dedicated to *Amblycerus virens* and *Amblycerus viridans*.

#### Etymology.

The specific name refers to the median line on dorsum.

## Supplementary Material

XML Treatment for Amblycerus virens

XML Treatment for Amblycerus virescens

XML Treatment for Amblycerus viridans

XML Treatment for Amblycerus viridis

XML Treatment for Amblycerus medialis
